# The Anti-Atherosclerotic Action of FFAR4 Agonist TUG-891 in ApoE–Knockout Mice Is Associated with Increased Macrophage Polarization towards M2 Phenotype

**DOI:** 10.3390/ijms22189772

**Published:** 2021-09-09

**Authors:** Anna Kiepura, Kamila Stachyra, Anna Wiśniewska, Katarzyna Kuś, Klaudia Czepiel, Maciej Suski, Magdalena Ulatowska-Białas, Marcin Surmiak, Rafał Olszanecki

**Affiliations:** 1 Chair of Pharmacology, Faculty of Medicine, Jagiellonian University Medical College, 16 Grzegorzecka Street, 31-531 Cracow, Poland; a.kiepura@uj.edu.pl (A.K.); kamila.stachyra@uj.edu.pl (K.S.); anna.niepsuj@uj.edu.pl (A.W.); katarzyna.1.kus@uj.edu.pl (K.K.); klaudia.czepiel@uj.edu.pl (K.C.); maciej.suski@uj.edu.pl (M.S.); 2 Department of Pathomorphology, Jagiellonian University Medical College, 16 Grzegorzecka Street, 31-531 Cracow, Poland; magdalena.bialas@uj.edu.pl; 3 Department of Internal Medicine, Faculty of Medicine, Jagiellonian University Medical College, 8 Skawińska Street, 31-066 Kraków, Poland; marcin.surmiak@uj.edu.pl

**Keywords:** free fatty acid receptors, FFAR4, inflammation, atherosclerosis, apoE-knockout mice, macrophages

## Abstract

**Background:** Over the past few years, a better understanding of the biology of G-protein coupled receptors (GPRs) has led to the identification of several receptors as novel targets for free fatty acids (FFAs). FFAR4 has received special attention in the context of chronic inflammatory diseases, including atherosclerosis, obesity and NAFLD, through to its anti-inflammatory effect. **Methods:** The present study investigates the influence of prolonged treatment with TUG-891-FFAR4 agonist on the development of atherosclerosis plaque in apoE-knockout mice, using morphometric and molecular methods. **Results:** TUG-891 administration has led to the reduction of atherosclerotic plaque size and necrotic cores in an apoE-knockout mice model. TUG-891-treated mice were administered subcutaneously at a dose of 20 mg/kg three times a week for 4 months. The FFAR4 agonist reduced the content of pro-inflammatory M1-like macrophages content in atherosclerotic plaques, as evidenced by immunohistochemical phenotyping and molecular methods. In atherosclerotic plaque, the population of smooth muscle cells increased as evidenced by α-SMA staining. We observed changes in G-CSF and eotaxin markers in the plasma of mice; changes in the levels of these markers in the blood may be related to macrophage differentiation. Importantly, we observed a significant increase in M2-like macrophage cells in atherosclerotic plaque and peritoneum. **Conclusions:** Prolonged administration of TUG-891 resulted in significant amelioration of atherogenesis, providing evidence that the strategy based on macrophage phenotype switching toward an M2-like activation state via stimulation of FFAR4 receptor holds promise for a new approach in the prevention or treatment of atherosclerosis.

## 1. Introduction

According to current paradigm, atherosclerosis is a systemic low-grade inflammatory disease characterized by progressive changes in arterial walls that involve many types of cells [1,2]. Dysfunction of endothelial cells, phenotypic changes of vascular smooth muscle cells (VSMC), as well as inflammatory stimulation and infiltration of lymphocytes and monocytes into the vessel wall were all reported to contribute to atherogenesis and formation of atherosclerotic plaques [1,3]. Studies in atherosclerosis underline a complex role of macrophages in local modulation of inflammation, development of plaques, and their rupture [4]. Macrophages can take many functional phenotypes; however, it is generally accepted that they are grouped closer to two extreme types, pro-inflammatory M1 and anti-inflammatory M2, both of which were identified to reside within the atherosclerotic plaque [5]. Growing evidence indicates that in-plaque M1 macrophages contribute to the atherosclerotic lesion progression towards a rupture-prone morphology, while M2 macrophages are linked to the resolution of local inflammation, reduction of plaque size, and its stabilization [5]. Thus, pharmacological methods of skewing macrophage polarization towards the M2 phenotype could represent an interesting new strategy for the treatment and /or prophylaxis of atherosclerosis and its complications [6].

Several G-protein coupled receptors (GPRs) for free fatty acids (FFARs) have been identified and partially characterized over recent years [6,7]. Among them the group of rhodopsin-like receptors including GPR40 (FFAR1), GPR41 (FFAR3), GPR43 (FFAR2), and GPR120 (FFAR4) have attracted the most attention [7]. By participating in anti-inflammatory effects, the FFAR4 became an interesting receptor in the studies of metabolic disorders and cardiovascular diseases [8]. It has been shown that next to adipocytes, hepatocytes, skeletal muscles, and epithelial cells, the FFAR4 is highly expressed in macrophages [9]. Oh et al. showed that activation of FFAR4 interfered with inflammatory LPS and TNF-α signaling in macrophages [9]. Interestingly, it has also been shown that FFAR4 stimulation may result in a beneficial phenotypical switch between M1/M2 macrophages infiltrating adipose tissue in obesity [9,10].

Studies exploring the anti-atherosclerotic potential of FFAR4 stimulation in vivo are very limited. There are few studies linking FFAR4 to several processes known to play a role in atherogenesis. Li et al. showed ω3 PUFAs mitigated vascular inflammation, arterial thrombus formation, and neointimal hyperplasia by interaction with FFAR4 in mice [11]. Kamata et al. in showed that EPA via FFAR4 activation reduced the expression of matrix metalloproteinase-9 (MMP-9) in the media of the aorta in a mouse model of aortic aneurysm [12]. Only recently, we have directly shown that activation of FFARs led to the inhibition of atherosclerosis in apoE−/− mice [13]. In this study, anti-atherosclerotic action of GW9508, a synthetic agonist of FFAR1 and FFAR4, was associated with a decrease of M1 macrophage content within the plaque [14]. It was the first study with the administration of a synthetic agonist of the FFARs in a mouse model of atherosclerosis, however its results are limited due to the non-selective action of GW9508 and its marked toxicity during prolonged administration [14]. The potency of GW9508 towards FFAR4 is 100 times lower compared to FFAR1 [13], while TUG-891 shows approximately 100 times greater potency and 10 times higher selectivity towards murine FFAR4 over FFAR1. Recently, TUG-891 has been found to up-regulate M2 and down-regulate M1 markers in rat macrophages in vitro [15].

In this study, we investigated whether the activation of FFAR4 by TUG-891 may inhibit formation of atherosclerotic lesions in apoE-knockout mice and elicit beneficial changes in plaque macrophage polarization.

## 2. Results

### 2.1. Body Weight and Plasma Levels of Lipids

TUG-891 was well tolerated by the animals and no toxic effects were observed during the experiment. The mean body weight of TUG-891-treated and the control animals did not differ (23.18 ± 1.15 g vs. 22.91 ± 1.33 g, *p* = 0.58). TUG-891 did not change the plasma levels of total cholesterol (TC), low-density lipoproteins (LDL), high-density lipoproteins (HDL) and triglycerides (TG) in apoE−/− mice (Table 1).

### 2.2. Effects of TUG-891 on Atherosclerosis

**Figure 1:**
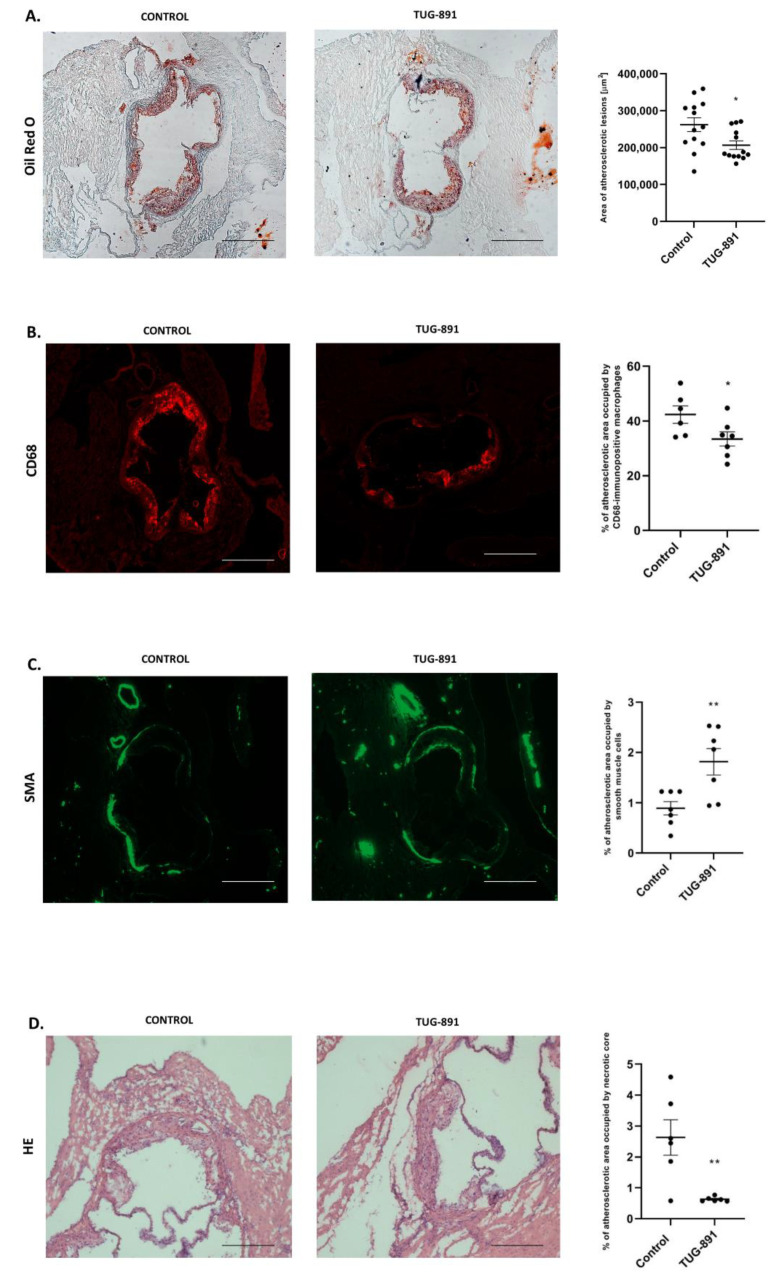
(**A**) Representative micrographs showing Oil Red O–stained lesions in control group and TUG-891-treated group. Atherosclerotic lesions area is measured by the cross-section method in control group and TUG-891-treated group (*p* < 0.05; *n*= 13 per group). (**B**,**C**) Macrophage infiltrated in the atherosclerotic lesion of TUG-891-treated apoE−/− mice. Immunohistochemical staining of aortic roots showing CD68-positive macrophages (red), α-SMA-positive (green). (mean ± SEM; * *p* < 0.05 as compared to apoE−/− mice; *n* = 6–7 per group). (**D**) Content of necrotic core in the atherosclerotic lesion of TUG-891-treated apoE−/− mice. Immunohistochemical staining showing necrotic core in atherosclerotic lesions of apoE−/− mice and TUG-891-treated apoE−/− mice (mean ± SEM; ** *p* < 0.005; *n* = 6–7 per group).

In the cross-section method, treatment with TUG-891 significantly reduced the area of atherosclerotic lesions in apoE−/− mice (262,108 µm^2^ ± 18,682 vs. 206,641 µm^2^ ± 11,532, *p* < 0.05; Figure 1A). Moreover, TUG-891 treatment decreased total macrophage content in atherosclerotic lesions, as evidenced by CD68 staining (42.36% ± 3.176 vs. 33.44% ± 2.576, *p* < 0.05; Figure 1B) and increased the population of smooth muscle cells, as evidenced by α-SMA staining in the fibromuscular cap (0.8942% ± 0.1322 vs. 1.816% ± 0.2598, *p* < 0.05; Figure 1C). Hematoxylin-eosin (HE) staining revealed that the TUG-891 administration markedly decreased necrotic cores in the plaques (2.631% ± 0.6299 vs. 0.5727% ± 0.03136 *p* < 0.05; Figure 1D).

### 2.3. Effects of TUG-891 on In-Plaque and Peritoneal Macrophage Phenotype

**Figure 2:**
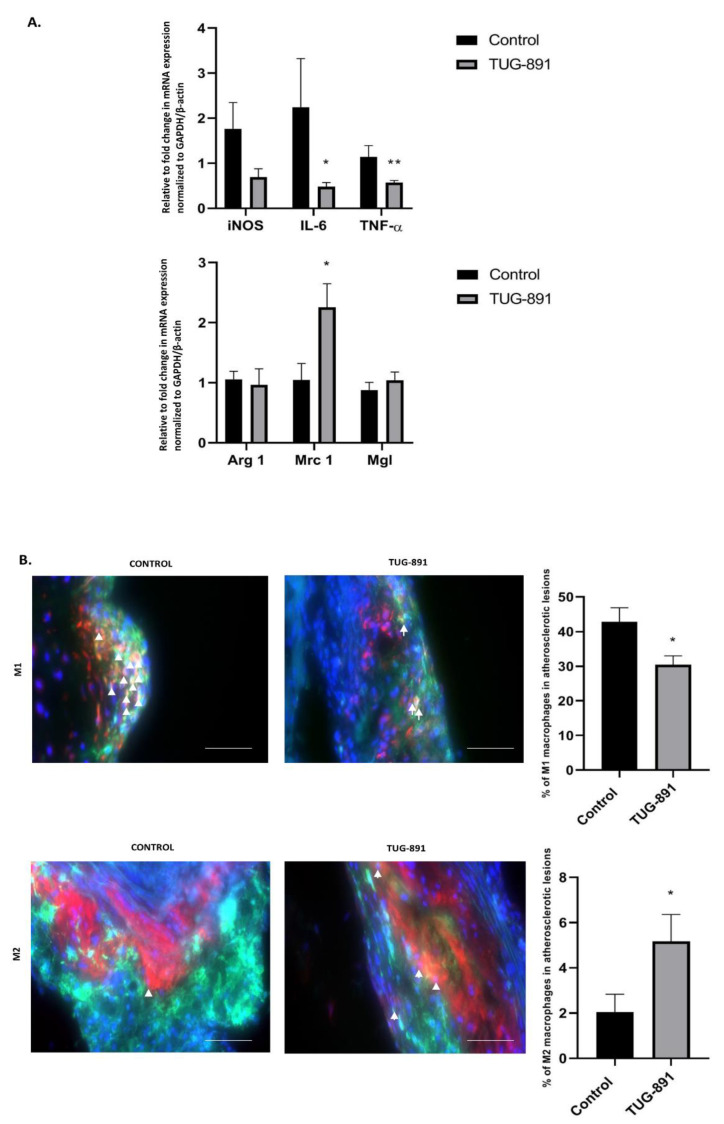
Macrophage phenotyping in atherosclerotic plaque. Validation of macrophages morphometry by RT-PCR analysis confirmed a trend toward reduction of M1 markers and no significant increase in M2 genes in the aorta of GW9508-treated apoE−/− mice, (**A**) (* *p* < 0.05; ** *p* < 0.005; *n* = 6–8 per group). Representative micrographs showing immunohistochemical staining of aortic roots from control and TUG-891-treated apoE-knockout mice. F4/80 (green), nitric oxide synthase 2 (iNOS)/arginase 1 (red) and 4′,6-diamidino-2-phenylindole (DAPI) (blue), confirming the respective macrophage phenotype (white arrows). Adjacent graphs represent mean ± SEM values, (**B**) (* *p* < 0.05; *n* = 5–7 per group).

**Figure 3:**
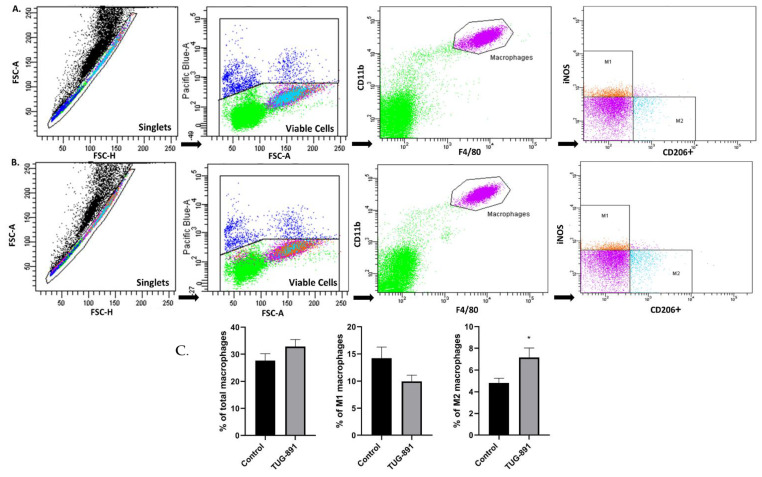
Macrophage phenotyping in peritoneal tissue. Representative flow cytometry histograms in peritoneal tissue from control and TUG-891-treated apoE-knockout mice. F4/80 and CD11b+ (**A**), F4/80, iNOS; CD11b+; F4/80 (**B**), CD206+; CD11b+; F4/80 (**C**). Adjacent graphs represent mean ± SEM values, (* *p* < 0.05; *n* = 10–14 per group).

The treatment with TUG-891 modified the phenotype of macrophages in the atherosclerotic plaques. TUG-891 administration significantly changed the aortic mRNA expression of genes associated with a pro-inflammatory M1-like phenotype (IL-6, TNF-α, iNOS). Among the genes associated with the M2-like phenotype TUG-891 increased the expression of Mrc1 (Figure 2A). Immunohistochemistry evaluation evidenced that the percentage of in-plaque iNOS-positive cells was lower (30.49% ± 2.552 vs. 42.80% ± 4.157, *p* < 0.05, Figure 2B), while percentage of arginase 1-positive cells increased (5.170% ± 1.182 vs. 2.037% ± 0.7960, *p* < 0.05; Figure 2B). The flow cytometric analysis of peritoneal macrophages revealed an increased percentage of M2 cells in TUG-891-treated animals (7.154% ± 0.8726 vs. 4.808% ± 0.4468, *p* < 0.05; Figure 3C). The population of peritoneal M1 macrophages decreased, however the change did not reach statistical significance (9.969% ± 1.156 vs. 14.23% ± 2.051, *p* = 0.0780; Figure 3B).

### 2.4. Effects of TUG-891 on Plasma Levels of Inflammatory Markers

**Figure 4:**
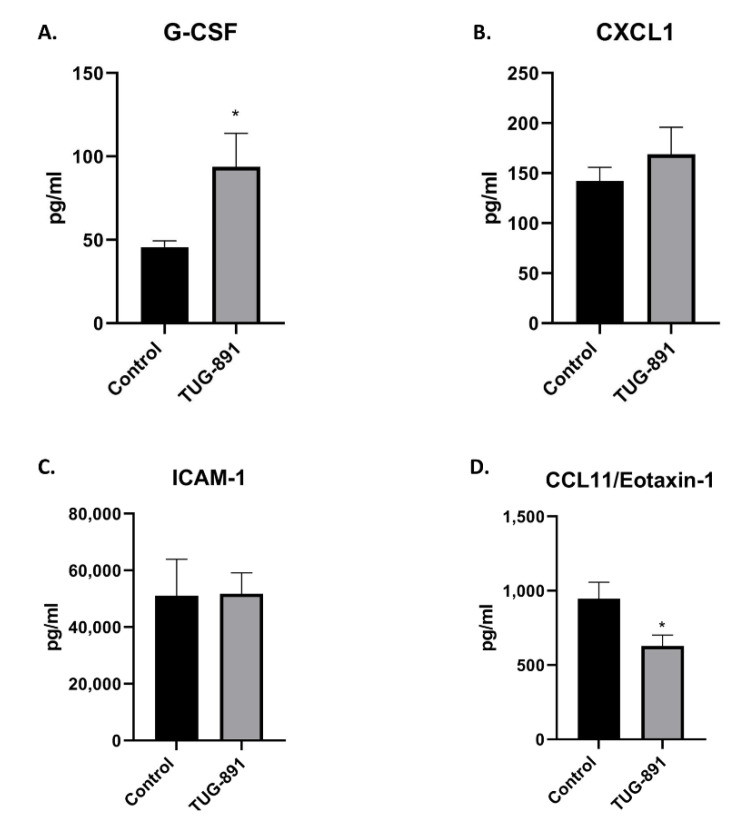
Plasma concentrations of G-CSF (**A**), CXCL1 (**B**), ICAM-1 (**C**), CCL11 (**D**) in control and TUG-891-treated apoE-knockout mice. Results are expressed as mean ± S.E.M; for *n* = 6–14 samples per group; * *p* < 0.05.

The plasma level of G-CSF was higher (93.82 pg/mL ± 20.17 vs. 45.74 pg/mL ± 3.685, *p* < 0.05), while concentration of CCL11 was lower in TUG-891-treated mice (628.4 pg/mL ± 74.06 vs. 946.1 pg/mL ± 112.1, *p* < 0.05 Figure 4A,D). The plasma levels of sICAM-1 and CXCL1 did not differ significantly between groups (sICAM-1: 51,792 pg/mL ± 7371 vs. 51,145 pg/mL ± 12,791, *p* = 0.4483; CXCL1: 169.1 pg/mL ± 26.77 vs. 142.3 pg/mL ± 13.59, *p* = 0.3940; Figure 4B,C).

## 3. Discussion

The major finding in this work is that inhibition of atherosclerosis by a selective FFAR4 agonist TUG-891 is associated with a significant shift in the polarization of macrophages in atherosclerotic plaques towards the M2 phenotype. It has been demonstrated that macrophages in plaques are highly pleiotropic and retain the capacity to shift their polarization [16]. Our data point to the role of FFAR4 in the modulation of macrophage phenotype and, in a more general context, also highlight the importance of in-plaque balance between different macrophage phenotype species as an important modulator of atherogenesis. It is recognized that alongside endothelial cells and vascular smooth muscle cells macrophages play a key role in atherogenesis, and overall macrophage functionality is critical to the balance between plaque progression and regression. It has been shown that the M2 phenotype macrophages can secrete anti-inflammatory factors and eferocyte dying cells, thus contribute to the repairment of damaged tissues [12]. Therefore, promoting the M2 polarization of macrophages at the expense of M1 may represent a promising new anti-atherosclerotic strategy, while pharmacological stimulation of FFAR4 appears to be an effective method to achieve this goal.

GW9508 was the first synthetic agonist for FFAR4 and FFAR1 [17] and remains a valuable tool in studies of the biological roles of FFA receptors in vitro, but its use in vivo may be may be compromised due to its poor tolerance in animals [14]. On the other hand, TUG-891 exhibits at least 1000-fold selectivity to FFAR4 over FFAR1 in assays on human cells (slightly less FFAR4 selectivity was noted in mouse models) and seems to be safe for animals [6]. An important advantage of TUG-891, in contrast to fatty acids, is that it does not undergo rapid metabolism in cells to secondary (often biologically active) metabolites, which actions may hinder the interpretation of the results [18,19]. Compared to GW9508, which reduced the amount of M1 macrophages in plaques, TUG-891 not only decreased the content of M1, but also increased significantly the content of M2 cells, which might be attributed to the increased selectivity towards FFAR4. Importantly, in our hands, TUG-891 in a dose selected based on the literature did not influence weight of animals or cause any toxic effects to apoE−/− mice. In our setting, the anti-atherosclerotic action of TUG-891 was not related to the changes in plasma lipids or typical for apoE−/− mice plasma inflammatory markers, such as sICAM-1 or CXCL1. On the other hand, in the context of the known role of G-CSF in macrophage differentiation, and recent reports about the possible role of eotaxin in differentiation of macrophages to the M2 phenotype [20], changes in blood levels of these two markers in TUG-891-treated apoE−/− seem to be an interesting lead for further research.

Our results suggest that FFAR4 activation can improve plaque stability, as the total number of macrophages in the plaques decreased in animals treated with TUG-891. This can be mainly attributed to the decrease in the content of M1 macrophages, which are typically dominant in plaques. However, we simultaneously evidenced smaller in absolute numbers but significant increase in the plaque population of M2 cells. Such changes were associated with the increase of the smooth muscle cells content in lesions, as evidenced by α-actin (SMA) staining. Along with the significant reduction of necrotic cores, this might indicate that TUG-891 not only ameliorates atherogenesis but also improves the plaque stability. It should be emphasized, however, that atherosclerotic lesions in the apoE−/− mice model are very stable and this model is not suitable for studying the effect of drugs on complications related to plaque rupture. Thus, the attractive hypothesis of anti-rupture effect of TUG-891 requires further investigation.

The modern paradigm of atherosclerosis indicates that atherosclerotic plaques expand as local manifestations in the course of a systemic, chronic, active, low-grade inflammatory process. It is important to note that the analysis of TUG-891-dependent changes of phenotype of peritoneal macrophages in apoE−/− mice may indicate systemic action of this compound on monocytes/macrophages. Our results are in line with the observations of Wang et al., who showed that FFAR4 activation in peritoneal macrophages could program them to the anti-inflammatory M2 phenotype [15]. Importantly, the role of FFAR4 in regulation of macrophage polarization could be also observed in vitro. Our recent preliminary results indicate that in the in vitro differentiation model of C57B6 mice, bone marrow-derived macrophages TUG-891 not only reduces the expression of M1 markers (iNOS, IL-6) but also increases the expression of M2 markers (Arg-1, MRC 1); moreover, its action is abolished by the FFAR4 antagonist AH-7614 [21]. The above data collectively appear to support the functional importance of the FFAR4 pathway in regulating the macrophage phenotype.

We can only speculate about the exact molecular mechanism(s) responsible for the regulation of macrophage polarization by FFAR4. Williams-Bey et al. showed that DHA acid may limit NLRP3 inflammasome activation via FFAR4 receptor; DHA treatment reduced the initial inflammasome priming step by suppressing the nuclear translocation of NF-κB and increased autophagy [10]. Other studies have shown that stimulation of FFAR4 on macrophages resulted in inhibition of the TLR2/3/4 and the TNF-α response pathways [3,7]. This could be related to the FFAR4-dependent inhibition of TAK-1 protein, which is a convergent transducer of TLR4 and TNF-α receptor pathways and NF-κB and MKK4/JNK signaling [13]. However, the exact mechanism(s) linking FFAR4 stimulation and macrophage polarization to M2 cells in apoE−/− mice require further elucidation.

The present study provides important in vivo evidence that stimulation of FFAR4 by the use of synthetic selective agonist TUG-891 significantly influences macrophage differentiation, favoring these cells to adopt the anti-inflammatory M2 phenotype, which is associated with anti-inflammatory and anti-atherogenic action. The primary role of macrophages in the progression of atherosclerosis in humans has been demonstrated. In this context, our results have significant translational potential and suggest the need for clinical trials using synthetic FFAR4 agonists. It may be that the stimulation of FFAR4 represents a promising strategy for the prevention and/or treatment of atherosclerosis, however the recognition of the exact mechanisms responsible for the FFAR-mediated modulation of the macrophage phenotype requires further studies.

### Limitations and Future Directions

Our study has several limitations. The most important result from necessary caution in transferring the results from animal models to the human situation. Moreover, in translational research, the possibility of orally administering a stable FFAR4 agonist should also be explored. Our studies also do not allow for the determination of the molecular mechanism linking FFAR4 stimulation and changes in the macrophage phenotype. Further in vitro and in vivo studies with additional methods (e.g., cytometry, molecular studies and single cell proteomics of macrophages isolated from atherosclerotic plaques, as well as the precise determination of the cellular localization of FFAR4 in the plaques by immunohistochemistry) would help to identify the candidates for such molecular link(s). Moreover, as a part of translational research, the influence of FFAR4 agonists on vulnerability of atherosclerotic plaques should be tested on relevant animal models (e.g., model of rupture of brachiocephalic artery in older apoE-deficient mice fed an HFD with or without angiotensin II infusion).

## 4. Materials and Methods

### 4.1. Animal Experiments

Male apoE-knockout mice (apoE−/−) on the C57BL/6J background were obtained from Charles River (Calco, Lecco, Italy). All animal procedures were approved by the Jagiellonian University Ethical Committee on Animal Experiments (No. 167/2018). The animals were kept on 12 h dark/12 h light cycles in air-conditioned rooms (22.5 ± 0.5 °C, 50 ± 5% humidity) with access to water ad libitum. The mice were put on a HFD (10% fat diet) made by Ssniff (E15122-34, S8435-E014, Soest, Germany) and treatment with compound TUG-891 at the age of 8 weeks for 16 weeks. Animals were randomly divided into two groups: the control group (apoE-knockout mice w/o treatment, on diet as above, *n* = 15) and TUG-891-treated mice (*n* = 15). TUG-891 (4-[(4-Fluoro-4′-methyl[1,1′-biphenyl]-2-yl)methoxy]-benzenepropanoic acid) (HY-100881, lot. 23051, Prospecta, Poland) was administered s.c. at a dose of 20 mg/kg 3 times (Monday, Wednesday, Friday) a week for 4 months. The daily dose of TUG-891 was chosen based on its effects described in other murine models [14,22,23]. At the age of 6 months the animals were injected with 1000 IU of fraxiparine i.p (Sanofi-Synthelabo, Paris, France) and killed in a chamber filled with carbon dioxide. Next, the blood was collected, and aortas, hearts, and livers were dissected.

### 4.2. Analysis of Atherosclerotic Plaque

The hearts with the ascending aorta were embedded in OCT compound (CellPath, Newtown, UK), snap frozen and sectioned (10 μm thickness) for histological and immunohistochemical analysis, according to the standardized cross-section protocol, as previously described [24]. The assessment was performed using Oil Red O and HE staining (O0625, lot. 093k3650, Sigma-Aldrich, St. Louis, MO) [25]. Immunohistochemistry for all macrophages and smooth muscles as well as polarization of macrophages was performed as described previously [14]. Immunohistochemistry for total macrophages and smooth muscles was performed with antibodies against CD68 (dilution 1:800; MCA1957, lot. 0812, Bio-Rad) and smooth muscle α-actin (SMA) (dilution 1:800; F3777, lot. 087M4798V, Sigma-Aldrich). Macrophage polarization was assessed with antibodies against F4/80 (dilution 1:100), nitric oxide synthase 2 (iNOS) (dilution 1:100, ab15323, lot. GR295447-1, Abcam), arginase 1 (dilution 1:200, ab91279, lot. GR3256056-1, Abcam), and 40,6-diamidino-2-phenylindole (DAPI; D9542, Sigma-Aldrich). The image was analyzed using LSM Image Browser software (Zeiss, Jena, Germany).

### 4.3. Real Time (RT)-PCR

Total RNA was isolated from the homogenized mouse aortas using the RNeasy Fibrous Tissue Mini Kit (74704, lot. 163042118, Qiagen, Germantown, MD, USA), according to the manufacturer’s instructions. The RNA concentration of each sample was measured at a wavelength of 260 nm (A260) in a Synergy H1 microplate reader (BioTek Instruments, Inc., Winooski, VT, USA). The purity of extracted total RNA was determined by the A260/A280 ratio. Then, cDNA was synthesized by the reverse transcription of 900 ng of total RNA from each sample, using a High-Capacity Reverse Transcription Kit (4374966, lot. 00895788, Applied Biosystems, Foster City, CA, USA). The cDNA was diluted ten-fold prior to PCR amplification. Real time quantitative PCR, using GoTaq^®^ qPCR Master Mix (A600A, lot. 0000463011, Promega, Madison, WI, USA), was carried out on the Bio-Rad CFX96 Touch™ Real-Time PCR System (Bio-Rad; Hercules, CA, USA). Primers for IL-6, TNF, ARG1, CD206/Mrc1 and MGL-1 were purchased from Real-TimePrimers.com (VMPS-3096, VMPS-6717, VMPS-407, VMPS-3915, Elkins Park, PA, USA) and primer for GAPDH and iNOS was purchased from Bio-Rad (qMmuCED0027497, lot.308468357; qMmuCID0023087, lot.281997755; qMmuCED0027505, lot. 357011322, Hercules, CA, USA). Analysis of the data was performed by the 2−ΔΔCt method using CFX Maestro Software (BioRad), and GAPDH/ β-actin expression was used as the internal control.

### 4.4. Biochemical Methods

Determination of CCL11, G-CSF, CXCL1, ICAM-1 concentrations in plasma was performed using Luminex micro beads fluorescent assays (R&D Systems, Inc., Minneapolis USA) and Luminex MAGPIX System (GDQN7xzB, Luminex Corp., Austin, TX, USA). Results were calculated from calibration curves and expressed in pg/mL, according to the manufacturer’s protocol [26]. The blood was collected from the right ventricle and centrifuged for 10 min, 1000 g at 4 °C. Plasma was harvested and stored in −80 °C until assayed. The plasma levels of total cholesterol, triglycerides, direct low-density lipoproteins (LDL) and high-density lipoproteins (HDL) were measured using an enzymatic method on a Cobas 8000 analyzer (Roche Diagnostics, Indianapolis, IN) as described previously [14]. Briefly, the Cobas system determines total cholesterol and HDL-cholesterol as well as TG by an enzymatic method with consecutive reactions by cholesterol esterase, cholesterol dehydrogenase and diaphorase and by lipoprotein lipase, lipoprotein dehydrogenase and diaphorase, respectively. LDL-C was directly measured by Roche LDL-Cholesterol plus 2nd generation reagent (homogenous enzymatic cholesterol assay by using cholesterol esterase and cholesterol oxidase/peroxidase).

### 4.5. Flow Cytometry

Peritoneal exudates cells were obtained by rinsing the peritoneal cavity with 5 mL of PBS. The cells were centrifuged, washed and counted. Equal numbers of cells were first stained with BD Horizon Fixable Viability Stain 450 (FVS450, lot. 8194969, BD Biosciences, San Diego, CA, USA) to excluded dead cells, according to the manufacturer’s instructions. Then the cells were preincubated with anti-mouse CD16/CD32 antibody (101302, lot. B264872, BioLegend, San Diego, CA, USA) to block FC receptors and labeled with PerCP-conjugated anti-mouse F4/80 (123126, lot. B331795, BioLegend, San Diego, CA, USA), FITC-conjugated anti-mouse/human CD11b (101206, lot. B260639, BioLegend, San Diego, CA, USA) and APC-conjugated anti-mouse CD206 (141708, lot. B318301, BioLegend, San Diego, CA, USA) antibodies. After surface staining, the cells were fixed and permeabilized using BD Cytofix/Cytoperm buffer (554714, lot. 9021740, BD Biosciences, San Diego, CA, USA) and then stained with PE-Cyanine7-coniugated anti-mouse NOS2 (25-5920-80, lot. 2017810, eBioscience, San Diego, CA, USA). Samples were acquired on a FACSCanto II flow cytometer (BD Biosciences, San Diego, CA, USA) and analyzed using FACSDiva software. F4/80 and CD11b were used as pan-macrophage markers, while NOS2 and CD206 were used as markers of M1 and M2 macrophages, respectively. F4/80-positive/CD11b-positive/NOS2-positive/CD206-negative cells were defined as M1 macrophages, whereas F4/80-positive/CD11b-positive/NOS2-negative/CD206-positive cells were identified as M2 macrophages.

### 4.6. Statistical Analysis

The results shown are mean ± SEM of multiple experiments. A comparison was carried out using Graphpad Prism and evaluated by 2-tailed Student’s *t*-test. All results are considered statistically significant with p values less than 0.05. Equality of variance and normality of the data were checked, and then the nonparametric Mann-Whitney’s U test (“cross-section”, biochemical methods and M1/M2 in plaque) or *t*-test (other methods) were used for statistical analysis of the data; *p* < 0.05 was considered as statistically significant.

## Figures and Tables

**Table 1:** Plasma levels of total cholesterol (TC), high-density lipoproteins (HDL), low-density lipoproteins (LDL), triglycerides (TG) in a control and TUG-891-treated group. Data presented as a mean ± SEM; *n* = 14 per group.

Group	Total Cholesterol [mmol/L]	HDL [mmol/L]	LDL [mmol/L]	TG [mmol/L]
apoE−/−	16.06 ± 2.55	1.28 ± 0.19	13.96 ± 2.03	1.02 ± 0.08
apoE−/− + TUG-891	16.29 ± 2.87	1.29 ± 0.18	14. 37 ± 2.56	1.005 ± 0.14

## Data Availability

All data are contained within this manuscript.

## Abbreviations

AH-7614	AH-7614-4-Methyl-N-9H-xanthen-9-yl-benzenesulfonamide
DAPI	4′:6-diamidino-2-phenylindole
DMSO	dimethyl sulfoxide
FBS	fetal bovine serum
FFAR4/GPR120	Free Fatty Acid Receptor 4
GADP	glyceraldehyde 3-phosphate dehydrogenase
HDL	high-density lipoproteins
HE	hematoxylin/eosin
iCAM-1	intercellular adhesion molecule 1
IL-1β	interleukin 1 beta
iNOS	inducible nitric oxide synthase
M1	classically activated macrophages
M2	alternatively activated macrophages
RT-PCR	reverse transcription polymerase chain reaction
SMA	smooth muscle actin
TC	total cholesterol
TG	triglyceride
TNF-α	tumor necrosis factor α
TUG-891	4-[(4-Fluoro-4′-methyl[1,1′-biphenyl]-2-yl)methoxy]-benzenepropanoic acid
